# Structural Retinal and Optic Nerve Changes in Prostate Cancer Patients Receiving Androgen Receptor Pathway Inhibitors: An OCT-Based In Vivo Analysis

**DOI:** 10.3390/diagnostics15131682

**Published:** 2025-07-01

**Authors:** Yasemin Bakkal Temi, Büşra Yılmaz Tuğan, İlkay Çıtakkul, Ece Baydar, Gözde Karaca, Sibel Balcı, Devrim Çabuk, Umut Kefeli, Nurşen Yüksel, Kazım Uygun

**Affiliations:** 1 Department of Internal Medicine and Medical Oncology, Faculty of Medicine, Kocaeli University, 41001 Kocaeli, Turkey; 2 Department of Ophthalmology, Faculty of Medicine, Kocaeli University, 41001 Kocaeli, Turkey; busrayilmaz87@hotmail.com (B.Y.T.);; 3 Department of Biostatistics and Medical Informatics, Kocaeli University, 41001 Kocaeli, Turkey

**Keywords:** prostate cancer, androgen receptor pathway inhibitors (ARPIs), optical coherence tomography (OCT), macular layers, retinal nerve fiber layer, minimum rim width

## Abstract

**Objective:** To conduct a comparative analysis of layer-by-layer macular thickness, peripapillary retinal nerve fiber layer (pRNFL), and minimum rim width (MRW) between the eyes of patients with prostate cancer undergoing treatment with androgen receptor pathway inhibitors (ARPIs) and those of age- and sex-matched healthy controls, with the aim of assessing the potential effects of ARPIs on retinal structure. **Methods:** In this prospective cross-sectional study, 80 eyes of 80 patients with ARPI-treated metastatic prostate cancer and 80 eyes of 80 age-matched healthy controls were evaluated using Heidelberg Spectralis optical coherence tomography (OCT). Layer-by-layer macular thickness, pRNFL, and MRW were measured and compared between groups. **Results:** Thickness in most segments of retinal layers and pRNFL, as well as all MRW values, were significantly lower in the ARPI-treated group than in the controls (*p* < 0.05). No significant differences were observed between groups in terms of age, visual acuity, intraocular pressure, central corneal thickness, or lens status. **Conclusions:** This study is the first to evaluate layer-by-layer macular thickness in patients with metastatic prostate cancer treated with ARPIs, revealing significant thinning in nearly all macular layers, pRNFL, and MRW. These findings suggest that ARPI therapy may induce neurodegenerative changes in retinal and optic nerve structures. Therefore, further research is warranted to assess the ocular safety of these therapeutic agents.

## 1. Introduction

Prostate cancer (PC) is the second most frequently diagnosed cancer worldwide [1]. Over the past five decades, screening has contributed to a 56% reduction in PC mortality [2]. Despite advancements in screening and prevention, PC remains a significant health concern, particularly for aging populations.

Prostate cancer treatment is determined by disease stage and severity. Androgen deprivation therapy (ADT) is the primary treatment for PC. Recent trials show intensified ADT with docetaxel or androgen receptor pathway inhibitors (ARPIs) improves progression-free survival and overall survival [3,4,5,6,7]. Current guidelines recommend ARPIs and/or chemotherapy with ADT as standard treatment for metastatic PC patients who can tolerate intensified therapy [8,9,10].

ARPIs are integral to the treatment of PC as they target the androgen axis. This category encompasses agents that directly bind to the androgen receptor (AR), thereby blocking its effects, such as enzalutamide and apalutamide, as well as CYP17 inhibitors, such as abiraterone acetate, which suppresses androgen production [11,12]. Although abiraterone does not directly target the AR, it indirectly inhibits this pathway by reducing androgen levels. The presence of AR in ocular tissues, such as the retina, prompts inquiries regarding the potential effects of ARPIs in these areas. Although the therapeutic role of ARPIs in managing prostate cancer is well-recognized, their potential implications for retinal health remain underexplored.

ARs are essential for mediating the effects of gonadal hormones and facilitating reproductive functions. Notably, AR is not confined to reproductive organs; it is also present in various tissues, including blood vessel cells and muscle cells, thereby influencing multiple body systems [13]. Androgens play a crucial role in maintaining cardiovascular health and promoting muscle development. Furthermore, the presence of AR has been identified in numerous ocular tissues, including the cornea, lens, iris, ciliary body, retina, lacrimal gland, meibomian gland, and conjunctiva [14,15,16]. Given the pivotal role of androgen receptors in both systemic and ocular physiology, it is essential to examine the effects of AR-targeted therapies on non-reproductive tissues. The retina, a highly specialized and hormone-sensitive structure, may be particularly vulnerable to such influences, necessitating a comprehensive investigation of these effects.

Modern imaging techniques enable a thorough segmentation analysis of individual retinal layers in patients receiving ARPIs. This technique aids in the early identification of potential structural alterations associated with therapy and may reveal the off-target effects of ARPIs on the retina. Specifically, examining ARPI-induced modifications in the macular and peripapillary regions is essential to understand early retinal involvement. In this regard, the assessment of macular thickness layer-by-layer, peripapillary retinal nerve fiber layer (pRNFL), and minimum rim width (MRW) could yield significant insights into the retinal impact of ARPIs.

To the best of our knowledge, no previous study has thoroughly evaluated retinal layer thickness in prostate cancer patients undergoing treatment with ARPIs. This lack of prior research underscores the need for comprehensive investigations of the potential ocular implications of ARPIs to ensure their safe application in clinical practice. This study aimed to assess the thickness of individual retinal layers, pRNFL, and MRW parameters in patients treated with ARPIs, and to compare these findings with those of healthy controls not exposed to ARPIs. Through this analysis, we sought to ascertain whether ARPI use is associated with early, identifiable changes in retinal structure, and whether these changes are clinically significant.

## 2. Materials and Methods

### 2.1. Patients

Between January 2024 and April 2025, a prospective evaluation was conducted at the Oncology and Ophthalmology Clinics of Kocaeli University Faculty of Medicine. The study included 80 eyes from 80 patients with metastatic prostate cancer undergoing ARPI treatment and 80 eyes from 80 age- and sex-matched healthy individuals. The research protocol was approved by the Kocaeli University Ethics Committee and followed the ethical standards of the Declaration of Helsinki. Informed consent was obtained from all participants prior to enrollment.

The exclusion criteria for participants in the patient cohort were as follows: cataract, corneal or lens opacities, glaucoma, general or hereditary retinal dystrophies, retinal detachment, degenerative myopia (≥6 D), age-related macular degeneration, and retinal diseases associated with diabetes or hypertension. Individuals diagnosed with metastatic prostate cancer and/or those who had undergone chemotherapy for other cancers were excluded from the study. In the control cohort, individuals with systemic conditions, such as cardiovascular, hematological, endocrine, neurological diseases, malignancies, cognitive disorders, or any ocular pathology, were excluded.

### 2.2. Ophthalmic Examination

All participants underwent thorough ophthalmic evaluations, which included assessments of best-corrected visual acuity (BCVA) and intraocular pressure measurement using Goldmann applanation tonometry, slit-lamp biomicroscopy, and gonioscopy with a Goldmann three-mirror lens. Perimetry was conducted using a Zeiss Humphrey Field Analyzer, and dilated fundus examination was performed. Vertical cup diameter was measured as the maximum distance between the superior and inferior boundaries of the optic cup.

### 2.3. Optical Coherence Tomography Imaging

Following pupil dilation, all eyes included in the study were subjected to spectral-domain optical coherence tomography (SD-OCT) imaging using the latest Heidelberg Spectralis OCT device equipped with the Glaucoma Module Premium Edition (GMPE; Heidelberg Engineering, Heidelberg, Germany). This imaging protocol comprehensively analyzed the entire macular layer, the pRNFL, and Bruch’s membrane opening–minimum rim width (BMO-MRW). A single ophthalmologist (BYT) meticulously reviewed all scans to detect segmentation errors, artifacts, or issues related to alignment and centration. Manual adjustments were not made to the scans. To ensure data integrity, only scans with a signal strength exceeding 40 were included in the analysis.

The GMPE software version 6.0 autonomously aligns the examination ring through its anatomical positioning system (APS), which identifies two predetermined anatomical landmarks: foveal center and BMO center. By linking these landmarks, the software established the foveal–BMO center axis. This axis was consistently maintained across successive scans, thereby ensuring precise sector-based data interpretation.

**Figure 1:**
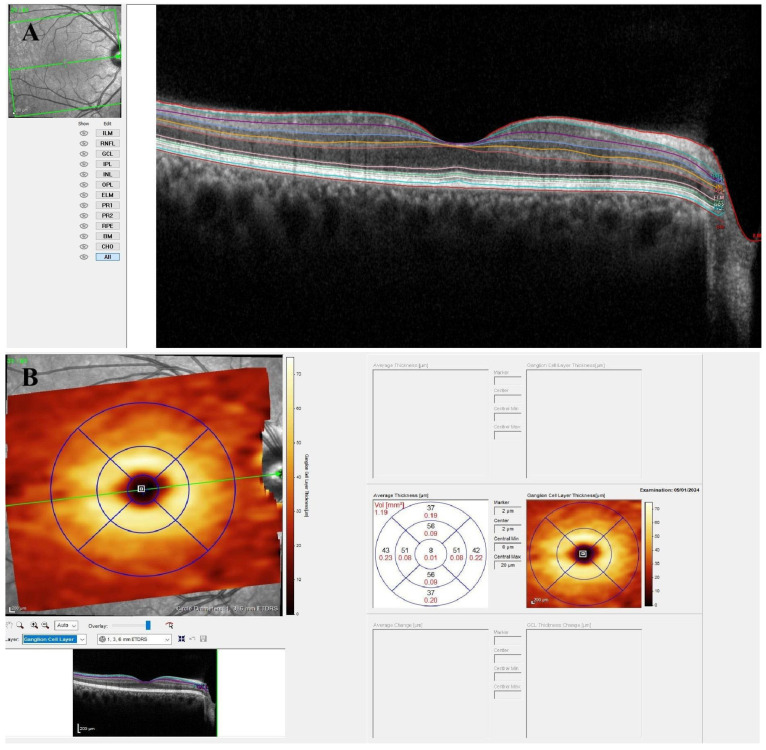
(**A**) An example of retinal layer segmentation in a healthy subject using Glaucoma Module Premium Edition (GMPE) software. (**B**) A retinal thickness analysis algorithm presenting mean thickness values (µm) for each of the nine regions defined by the Early-Treatment Diabetic Retinopathy Study (ETDRS) circle. Ganglion cell-layer measurements in a healthy subject are shown as an example.

To assess the average thickness of various retinal layers, we employed the retinal thickness map technique within the nine regions delineated by the Early-Treatment Diabetic Retinopathy Study (ETDRS) circle. This circle comprised three concentric rings with diameters of 1 mm (inner), 3 mm (middle), and 6 mm (outer), all centered on the fovea. The middle and outer rings were further subdivided into four quadrants: superior, nasal, inferior, and temporal. Central foveal thickness was determined by averaging all measurements within the 1 mm central ring. Using the GMPE software’s layer-by-layer segmentation tool, we evaluated the thickness of seven macular regions for the following layers: (1) macular nerve fiber layer (mNFL), (2) ganglion cell layer (GCL), (3) inner plexiform layer (IPL), (4) inner nuclear layer (INL), (5) outer plexiform layer (OPL), (6) outer nuclear layer (ONL), and (7) retinal pigment epithelium (RPE). Figure 1 illustrates the segmentation and regional thickness analysis of the GCL, based on the ETDRS grid.

**Figure 2:**
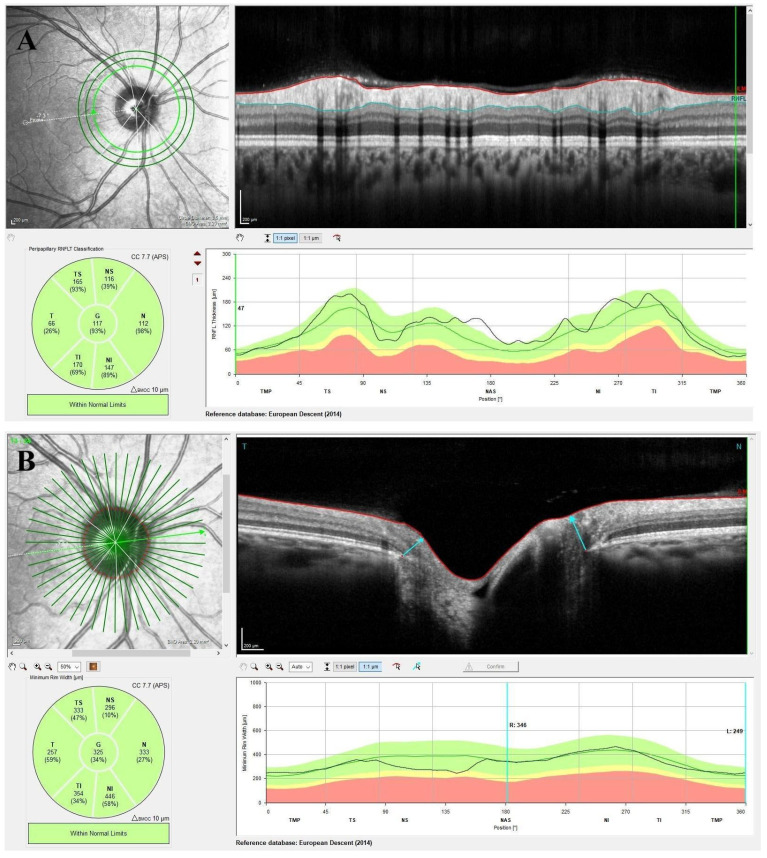
(**A**) Retinal nerve fiber layer (RNFL) thickness analysis in a healthy subject using a 3.5 mm-diameter peripapillary scan circle. (**B**) Bruch’s membrane opening–minimum rim width (BMO-MRW) (blue arrow) analysis in a healthy subject for optic nerve head rim evaluation.

pRNFL thickness was measured automatically from the outer edge of the RNFL to the inner edge of the internal limiting membrane (ILM) and analyzed in six sectors around the optic disk within a 3.5 mm circular region (superotemporal, superonasal, nasal, inferonasal, inferotemporal, and temporal). Figure 2A shows a representative example of the RNFL thickness analysis in a healthy subject. The BMO-MRW procedure of GMPE automatically identified the boundaries of the BMO before capturing 24 radial line B scans (totaling 48 BMO points) centered on the ONH. The BMO-MRW parameter was calculated using the shortest distance between the BMO points and the internal limiting membrane. Figure 2B shows the BMO-MRW analysis protocol and the sectoral evaluation. For analysis, the BMO area was divided into six sections, and each section averaging the corresponding BMO-MRW data and comparing it to age-matched controls. All measurements were taken in the morning to reduce the impact of diurnal variations.

### 2.4. Statistical Analysis

All statistical analyses were performed using the IBM SPSS Statistics 20.0 (IBM Corp., Armonk, NY, USA) and MedCalc 14.0. The assumption of normality was evaluated using the Shapiro–Wilk test. In cases where the data were not normally distributed, numerical variables were presented as medians with interquartile ranges (25th–75th percentiles). Categorical variables are summarized as counts and percentages. Comparisons of numerical variables between the groups were conducted using the Mann–Whitney U test. Associations between categorical variables were analyzed using the chi-squared test. Statistical significance was set at *p* < 0.05.

## 3. Results

Table 1 provides a summary of the demographic and clinical characteristics of both the patient and control groups. The median age was 70 (IQR: 62–75.75) years in the patient group and 70 (IQR: 60–72) years in the control groups. No significant differences were found between the two groups regarding age, BCVA, IOP, CCT and lens status (*p* = 0.136, *p* = 0.300, *p* = 0.079, *p* = 0.179 and *p* = 0.059, respectively). All patients demonstrated normal results on the Humphrey visual field test with no evidence of functional visual field loss at the time of assessment.

For the patient group, the treatment duration was 15 months (IQR: 9–18), and the baseline PSA level was 46 ng/mL (IQR: 11.25–184). In the study group, 17.5% of the patients (*n* = 14) were treated with abiraterone, while 82.5% (*n* = 66) received enzalutamide, totaling 80 patients.

Layer-by-layer macular thickness parameters are listed in Table 2. Most NFL, GCL, IPL, INL, OPL, ONL, and RPE thickness parameters in the patient group were significantly lower than those in the control group.

The pRNFL and BMO-MRW parameters demonstrated in Table 3. Most pRNFL thickness and all MRW parameters were significantly lower in the patient group than in the control group.

A subgroup analysis comparing the abiraterone and enzalutamide groups indicated no statistically significant differences in the OCT-based structural and layer-specific parameters.

Subgroup analysis was performed to evaluate the relationship between the duration of ARPI treatment and alterations in the retinal structure. Participants were categorized into two groups: those with treatment duration of less than 15 months (*n* = 38) and those with treatment duration of 15 months or more (*n* = 42). Statistically significant thinning was detected in the ≥15-month group across several macular sublayers and in the nasal sectors of the peripapillary RNFL and MRW. Furthermore, Spearman’s correlation analysis identified weak yet statistically significant negative correlations between the treatment duration and the thickness of the outer inferior IPL (r = −0.231, *p* = 0.039) and outer nasal ONL (r = −0.270, *p* = 0.015).

## 4. Discussion

This study represents the first layer-by-layer analysis of macular thickness in patients with metastatic prostate cancer treated with androgen receptor pathway inhibitors compared with age-matched healthy controls. Our quantitative analysis demonstrated a statistically significant thinning across most of the sectors in all macular layers in the ARPI-treated group. Similarly, we observed significant thickness reductions in most sectors of the PRNFL and all sectors of MRW compared to controls.

Recent investigations into androgen signaling in prostate cancer have facilitated the development of ARPIs such as abiraterone, enzalutamide, apalutamide, and darolutamide. Nevertheless, when ARPIs are administered in conjunction with standard ADT, significant adverse effects are observed [17]. Among these, anemia, osteoporosis, fatigue, weight gain, hot flashes, diabetes, hypertension, and increased cardiovascular risk have been reported [17,18]. Adverse reactions associated with the central nervous system (CNS), particularly fatigue and decreased cognitive function, are frequently reported with ARPIs [19,20]. Pilon et al. documented that the overall risk of CNS events was 7.3% for abiraterone and 9.1% for enzalutamide [19]. Enzalutamide’s ability to cross the blood–brain barrier may contribute to increased neurological events such as headache, seizures, and posterior reversible encephalopathy [21,22]. Given the prevalence of adverse neurological effects, it is imperative to assess whether these agents also affect the neurological and vascular structures of the retina. Examining the impact of ARPIs on the macula and optic nerve head could yield further insights into their broader neurotoxic profiles and aid in elucidating the mechanisms underlying CNS-related side effects. The observed thinning of the macular layer may indicate retinal involvement in the neurotoxic profile of ARPIs, thereby supporting the hypothesis that these agents affect ocular structures associated with the central nervous system.Despite notable thinning in structural layers, such as the pRNFL and GCL, the lack of visual field deficits may suggest preperimetric retinal damage, which is characteristic of early glaucomatous alterations [23].

Furthermore, the subgroup analysis based on treatment duration indicated that patients undergoing ARPI therapy for ≥15 months demonstrated significantly greater thinning in several retinal parameters, including the NFL outer superior, GCL outer superior, IPL outer inferior, and ONL outer inferior. These findings suggest a potential cumulative neurotoxic effect of prolonged ARPI exposure. Although the cross-sectional design precludes definitive conclusions regarding causality, the results support the hypothesis that extended ARPI use contributes to progressive structural alterations in the retina.

ARs have been identified in various structures within the human eye [14,16]. These findings imply that androgen blockade may directly affect ocular health. It has been documented that luteinizing hormone-releasing hormone (LHRH) analogs may elevate the risk of dry eye and conjunctival irritation. In addition, although infrequent, blurred vision has been reported during ARPI treatment [24,25]. Additionally, elevated levels of AR have been detected in glaucomatous astrocytes, suggesting a potential role of androgen metabolism in contributing to optic nerve damage in glaucoma [26]. The mechanisms underlying the potential neuroprotective or neurodegenerative effects of androgens remain inadequately understood. The presence of AR in the retina indicates that it may serve as a target for androgenic regulation. The inhibition of these receptors through ARPI therapy can disrupt normal retinal cellular functions, potentially resulting in structural alterations in the retina and macula. In our study, such alterations were evidenced by significant thinning of the pRNFL and multiple macular layers in the ARPI-treated group compared to the controls.

Previous studies have explored the effects of elevated androgen levels on retinal structures, focusing primarily on populations exposed to endogenous or exogenous testosterone. For example, an increase in the thickness of the superior RNFL has been documented in women diagnosed with polycystic ovary syndrome who also have a body mass index > 30 kg/m^2^. This observation suggests a potential trophic effect of androgens on retinal nerve fibers [27]. Similarly, in female-to-male transgender individuals administered supraphysiological doses of testosterone, an increase in both macular and RNFL thicknesses has been observed. Furthermore, a positive correlation was observed between perifoveal thickening and serum testosterone levels [28]. In contrast, our findings revealed significant thinning across all macular layers and pRNFL in patients undergoing ARPI-based treatment for prostate cancer. These contrasting results underscore the potential neurotrophic effects of androgens on retinal tissues. Androgen deprivation, particularly ARPI therapy, may lead to neurodegenerative changes. This suggests a possible association between the inhibition of the androgen receptor pathway and structural neurodegeneration in the retina. This implies that androgenic signaling is essential for maintaining the architecture of the retina and optic nerve. Given the critical supportive role of RPE in retinal physiology, damage to this layer may also trigger secondary degeneration in the inner retinal structures [29,30].This supports the possibility that both direct and indirect mechanisms contribute to retinal thinning observed in ARPI-treated patients [31].

Although our study has notable strengths, it is crucial to recognize its limitations. Owing to the cross-sectional design and the absence of baseline retinal thickness measurements prior to the initiation of ARPI, we were unable to definitively attribute the observed structural changes to treatment. Additionally, the lack of longitudinal follow-up data constrains the evaluation of retinal change progression over time. Moreover, the simultaneous use of androgen deprivation therapy and the inclusion of patients treated with both abiraterone and enzalutamide complicates the isolation of the effects of individual agents. Finally, the unequal distribution of patients between the abiraterone and enzalutamide groups may have reduced the statistical power to detect potential differences between the two ARPIs.

In conclusion, this study demonstrated that patients with metastatic prostate cancer undergoing treatment with ARPIs exhibited significant reductions in layer-by-layer macular thickness, pRNFL thickness, and MRW compared with healthy controls. These findings indicate the potential ocular effects of ARPI therapy. Consequently, comprehensive ophthalmologic evaluations and regular follow-up assessments are recommended to facilitate the early detection and management of possible retinal changes. Further investigation is necessary to ascertain whether ARPIs are an independent risk factor for retinal damage. Moreover, the potential variations in ocular toxicity profiles among different agents require examination in larger patient cohorts, as the abiraterone group demonstrated a non-significant trend towards increased thinning across several parameters.

## Figures and Tables

**Table 1:** A comparison of the clinical and demographic characteristics between the groups.

	Patient Group (*n* = 80)	Control Group (*n* = 80)	*p*
Age (years)	70 (62–75.75)	70 (60–72)	0.136 ^a^
BCVA (logMAR)	0.1 (0.1–0.3)	0.1 (0.1–0.3)	0.300 ^a^
IOP	14 (13–14.75)	13.5 (11–15.75)	0.079 ^a^
CCT	534.5 (526–541.75)	531 (525–539)	0.179 ^a^
Lens status			0.054 ^b^
Phakic	40 (50.0%)	53 (66.3%)	
Pseudophakic	40 (50.0%)	27 (33.7%)	
Treatment characteristics	Abiraterone: 14 (17.5%), Enzalutamide: 66 (82.5%)	-	

Data are presented as median (IQR). BCVA: best-corrected visual acuity; IOP: intraocular pressure; CCT: Central corneal thickness. ^a^ Mann–Whitney U test. ^b^ Chi-square test *p* < 0.05 shown in boldface.

**Table 2:** A comparison of layer-by-layer thickness parameters in different sectors of macular region between the groups.

	Patient Group (*n* = 80)	Control Group (*n* = 80)	*p* ^a^
Macular nerve fiber layer thickness (mNFL), µm			
mNFL outer superior	22 (16–37.75)	29 (24–36)	**0.003**
mNFL outer inferior	24 (19.25–43.75)	29 (24.25–34.75)	0.432
mNFL outer temporal	19 (17–21)	19 (17–21.75)	0.624
mNFL outer nasal	19 (18–21)	28.5 (16–59)	0.058
mNFL inner superior	30 (24–32)	64 (55–74)	**<0.001**
mNFL inner inferior	27 (22–32.75)	32 (25.25–35)	**0.003**
mNFL inner temporal	18 (16–20)	31 (30–33)	**<0.001**
mNFL inner nasal	24 (19.25–34)	26 (24.25–27.75)	0.176
mNFL central	17 (14–18)	29 (26–32)	**<0.001**
Ganglion cell layer thickness (GCL), µm			
GCL outer superior	30 (25–48)	48 (41–54)	**<0.001**
GCL outer inferior	27 (23–52)	45 (41–48)	**<0.001**
GCL outer temporal	28.5 (22.25–35)	41 (34–46)	**<0.001**
GCL outer nasal	33 (29–37.75)	47 (44–51)	**<0.001**
GCL inner superior	44 (39.25–48.75)	52.5 (44.25–56)	**<0.001**
GCL inner inferior	45 (39–49)	52 (43.5–56)	**<0.001**
GCL inner temporal	40 (33.25–48)	44.5 (40–48.75)	**0.011**
GCL inner nasal	45 (40.25–50)	45.5 (40–51)	0.973
GCL central	36.5 (24–41.75)	45 (40–48)	**<0.001**
Inner plexiform layer thickness (IPL), µm			
IPL outer superior	26 (22–42)	38 (33–40)	**<0.001**
IPL outer inferior	27.5 (20–41.75)	34.5 (32–37)	0.059
IPL outer temporal	31.5 (27–36)	33 (29–35)	0.762
IPL outer nasal	28 (24–34)	34 (29–35)	**<0.001**
IPL inner superior	34 (30–36)	41 (35–44)	**<0.001**
IPL inner inferior	33 (30–34)	41 (38–44)	**<0.001**
IPL inner temporal	34 (29.25–36)	36 (32.25–41)	**<0.001**
IPL inner nasal	32 (28–38)	36 (32–42)	**<0.001**
IPL central	32.5 (26.25–37)	39 (37–40.75)	**<0.001**
Inner nuclear layer thickness (INL), µm			
INL outer superior	33 (27–39)	35 (32–40)	**<0.001**
INL outer inferior	33 (32–35)	33.5 (26–39)	**<0.001**
INL outer temporal	30 (28–33.75)	37 (35–38)	**<0.001**
INL outer nasal	32 (30–35)	36.5 (32–38)	**<0.001**
INL inner superior	34 (32–35)	42 (38–45)	**<0.001**
INL inner inferior	32 (31–34)	41 (38–44)	**<0.001**
INL inner temporal	33 (31.25–35)	36 (33–41)	**<0.001**
INL inner nasal	32 (31–34)	39 (35–43)	**<0.001**
INL central	33 (32–35)	35 (28.5–40)	**0.016**
Outer plexiform layer thickness (OPL), µm			
OPL outer superior	26.5 (24–29.75)	29 (28–31)	0.160
OPL outer inferior	26 (24–29)	27 (25–29)	0.142
OPL outer temporal	26 (25–28)	27 (25.25–29)	**<0.001**
OPL outer nasal	28 (25–29)	28 (26.25–31)	**<0.001**
OPL inner superior	27 (25–28)	31 (29–37)	**<0.001**
OPL inner inferior	26 (25–28)	31 (28–36.75)	**<0.001**
OPL inner temporal	27 (25–29)	31 (27–35)	**<0.001**
OPL inner nasal	28 (25–29)	31 (28–35)	**<0.001**
OPL central	27 (25–28)	33.5 (28–40.75)	**<0.001**
Outer nuclear layer thickness (ONL), µm			
ONL outer superior	60 (50.25–70.25)	68 (65–69)	**<0.001**
ONL outer inferior	55 (47–70)	58 (55–61.75)	0.199
ONL outer temporal	53 (48–60)	59 (55–63)	**<0.001**
ONL outer nasal	58 (53.25–62)	59 (46.25–74)	0.827
ONL inner superior	59 (55–61)	65.5 (55–75.75)	**<0.001**
ONL inner inferior	58 (55–60.75)	70 (60–77)	**<0.001**
ONL inner temporal	59 (55–62)	69.5 (60–79.75)	**<0.001**
ONL inner nasal	58 (55–63)	64 (56.25–73.75)	**0.001**
ONL central	59 (55–62)	85.5 (75.25–95)	**<0.001**
Retina pigment epithelium thickness (RPE), µm			
RPE outer superior	13 (12–14)	16 (15–17)	**<0.001**
RPE outer inferior	12 (11–13)	15 (14–16)	**<0.001**
RPE outer temporal	12 (11–13)	15 (15–16)	**<0.001**
RPE outer nasal	13 (11–15)	15 (15–16)	**<0.001**
RPE inner superior	14 (13–15)	16 (15–16.75)	**<0.001**
RPE inner inferior	14 (13–15)	16 (15–17)	**<0.001**
RPE inner temporal	14 (13–15)	16 (15–17)	**<0.001**
RPE inner nasal	13.5 (13–15)	16 (15–16)	**<0.001**
RPE central	15 (14–16)	16 (15–17)	0.064

mNFL, macular nerve fiber layer; GCL, ganglion cell layer; IPL, inner plexiform layer; INL, inner nuclear layer; OPL, outer plexiform layer; ONL, outer nuclear layer; RPE, retina pigment epithelium. Data are presented as median (IQR). ^a^ Mann–Whitney U test. *p* < 0.05 shown in boldface.

**Table 3:** A comparison of thickness parameters in different sectors of pRNFL and MRW between the groups.

	Patient Group (*n* = 80)	Control Group (*n* = 80)	*p* ^a^
Peripapillary retinal nerve fiber layer thickness (pRNFL), µm			
pRNFL-TS	126 (119–137.5)	128 (116–143)	0.777
pRNFL-NS	106.5 (95–120)	120 (102.5–139.5)	**<0.001**
pRNFL-N	78.5 (68.5–95)	86.5 (79–89)	0.160
pRNFL-NI	120.5 (104.25–134.25)	132 (122–135)	**0.005**
pRNFL-TI	135 (124.25–138)	137 (123.25–155.5)	**0.047**
pRNFL-T	75 (67.25–86.5)	85 (79–89)	**0.001**
pRNFL-G	103 (97.25–110)	117 (112–121)	**<0.001**
Minimum rim width (MRW), µm			
MRW-TS	320 (260–374.25)	397 (355–425)	**<0.001**
MRW-NS	356.5 (265.75–407.75)	421 (353–521)	**<0.001**
MRW-N	324.5 (266.5–396.5)	410.50(344.50–433.00)	**<0.001**
MRW-NI	403.5 (357.75–452.5)	469.00(409.00–522.00)	**<0.001**
MRW-TI	352 (287.5–411)	421.00(325.00–449.00)	**<0.001**
MRW-T	249.5 (219.5–345.75)	250.00(195.00–285.00)	**0.035**
MRW-G	338.5 (285.75–365.75)	388.5 (316.25–424.75)	**<0.001**

G indicates global; pRNFL, peripapillary retinal nerve fiber layer; MRW, minimum rim width; N, nasal; NI, nasal inferior; NS, nasal superior; T, temporal; TI, temporal inferior; TS, temporal superior. Data are presented as median (IQR). ^a^ Mann–Whitney U test. *p* < 0.05 shown in boldface.

## Data Availability

Dataset available on request from the authors.The data are not publicly available due to patient confidentiality and ethical restrictions.

## References

[1] Filho A.M., Laversanne M., Ferlay J., Colombet M., Piñeros M., Znaor A., Parkin D.M., Soerjomataram I., Bray F. (2025). The GLOBOCAN 2022 cancer estimates: Data sources, methods, and a snapshot of the cancer burden worldwide. Int. J. Cancer.

[2] Goddard K.A.B., Feuer E.J., Mandelblatt J.S., Meza R., Holford T.R., Jeon J., Lansdorp-Vogelaar I., Gulati R., Stout N.K., Howlader N. (2025). Estimation of Cancer Deaths Averted from Prevention, Screening, and Treatment Efforts, 1975–2020. JAMA Oncol..

[3] James N.D., Sydes M.R., Clarke N.W., Mason M.D., Dearnaley D.P., Spears M.R., Ritchie A.W.S., Parker C.C., Russell J.M., Attard G. (2016). Addition of docetaxel, zoledronic acid, or both to first-line long-term hormone therapy in prostate cancer (STAMPEDE): Survival results from an adaptive, multiarm, multistage, platform randomised controlled trial. Lancet.

[4] Fizazi K., Tran N., Fein L., Matsubara N., Rodriguez-Antolin A., Alekseev B.Y., Özgüroğlu M., Ye D., Feyerabend S., Protheroe A. (2017). Abiraterone plus Prednisone in Metastatic, Castration-Sensitive Prostate Cancer. N. Engl. J. Med..

[5] Davis I.D., Martin A.J., Stockler M.R., Begbie S., Chi K.N., Chowdhury S., Coskinas X., Frydenberg M., Hague W.E., Horvath L.G. (2019). Enzalutamide with Standard First-Line Therapy in Metastatic Prostate Cancer. N. Engl. J. Med..

[6] Chi K.N., Agarwal N., Bjartell A., Chung B.H., Pereira de Santana Gomes A.J., Given R., Juárez Soto Á., Merseburger A.S., Özgüroğlu M., Uemura H. (2019). Apalutamide for Metastatic, Castration-Sensitive Prostate Cancer. N. Engl. J. Med..

[7] Smith M.R., Hussain M., Saad F., Fizazi K., Sternberg C.N., Crawford E.D., Kopyltsov E., Park C.H., Alekseev B., Montesa-Pino Á. (2022). Darolutamide and Survival in Metastatic, Hormone-Sensitive Prostate Cancer. N. Engl. J. Med..

[8] Schaeffer E.M., Srinivas S., Adra N., An Y., Barocas D., Bitting R., Bryce A., Chapin B., Cheng H.H., D’Amico A.V. (2022). NCCN Guidelines^®^ Insights: Prostate Cancer, Version 1.2023: Featured updates to the NCCN guidelines. J. Natl. Compr. Cancer Netw..

[9] Parker C., Castro E., Fizazi K., Heidenreich A., Ost P., Procopio G., Tombal B., Gillessen S. (2020). Prostate cancer: ESMO Clinical Practice Guidelines for diagnosis, treatment and follow-up. Ann. Oncol..

[10] Cornford P., van den Bergh R.C.N., Briers E., van den Broeck T., Cumberbatch M.G., De Santis M., Fanti S., Fossati N., Gandaglia G., Gillessen S. (2021). EAU-EANM-ESTRO-ESUR-SIOG Guidelines on Prostate Cancer. Part II—2020 Update: Treatment of Relapsing and Metastatic Prostate Cancer. Eur. Urol..

[11] Tran C., Ouk S., Clegg N.J., Chen Y., Watson P.A., Arora V., Wongvipat J., Smith-Jones P.M., Yoo D., Kwon A. (2009). Development of a second-generation antiandrogen for treatment of advanced prostate cancer. Science.

[12] Thakur A., Roy A., Ghosh A., Chhabra M., Banerjee S. (2018). Abiraterone acetate in the treatment of prostate cancer. Biomed. Pharmacother..

[13] Lucas-Herald A.K., Alves-Lopes R., Montezano A.C., Ahmed S.F., Touyz R.M. (2017). Genomic and non-genomic effects of androgens in the cardiovascular system: Clinical implications. Clin. Sci..

[14] Rocha E.M., Wickham L.A., Ada Silveira L., Krenzer K.L., Yu F.-S., Toda I., Sullivan B.D., ASullivan D. (2000). Identification of androgen receptor protein and 5alpha -reductase mRNA in human ocular tissues. Br. J. Ophthalmol..

[15] Wickham L.A., Gao J., Toda I., Rocha E.M., Ono M., Sullivan D.A. (2000). Identification of androgen, estrogen and progesterone receptor mRNAs in the eye. Acta Ophthalmol. Scand..

[16] Gupta P.D., Johar K., Nagpal K., Vasavada A.R. (2005). Sex hormone receptors in the human eye. Surv. Ophthalmol..

[17] Nguyen P.L., Alibhai S.M.H., Basaria S., D’Amico A.V., Kantoff P.W., Keating N.L., Penson D.F., Rosario D.J., Tombal B., Smith M.R. (2015). Adverse effects of androgen deprivation therapy and strategies to mitigate them. Eur. Urol..

[18] Leong D.P., Cirne F., Pinthus J.H. (2025). Cardiovascular Risk in Prostate Cancer. Cardiol. Clin..

[19] Pilon D., Behl A.S., AEllis L., Robitaille M.-N., Lefebvre P., Dawson N.A. (2017). Assessment of Real-World Central Nervous System Events in Patients with Advanced Prostate Cancer Using Abiraterone Acetate, Bicalutamide, Enzalutamide, or Chemotherapy. Am. Health Drug Benefits.

[20] Cao B., Kim M., Reizine N.M., Moreira D.M. (2023). Adverse Events and Androgen Receptor Signaling Inhibitors in the Treatment of Prostate Cancer: A Systematic Review and Multivariate Network Meta-analysis. Eur. Urol. Oncol..

[21] Slovin S., Clark W., Carles J., Krivoshik A., Park J.W., Wang F., George D. (2018). Seizure rates in enzalutamide-treated men with metastatic castration-resistant prostate cancer and risk of seizure: The UPWARD study. JAMA Oncol..

[22] Crona D.J., Whang Y.E. (2015). Posterior reversible encephalopathy syndrome induced by enzalutamide in a patient with castration-resistant prostate cancer. Investig. New Drugs.

[23] Ozaltun O.A., Koz O.G., Yarangumeli A.A. (2025). Evaluation of macula ganglion cell analysis and retinal nerve fiber layer thickness in preperimetric glaucoma, early stage glaucoma and healthy individuals. Photodiagnosis Photodyn. Ther..

[24] Li W., Li X., Cui F., Xu Z., Dong N., Li C., Shi W.-Q. (2021). The Ocular Surface Characteristics in Prostate Cancer Patients Treated with Androgen Deprivation Therapy. Dis. Markers.

[25] Chien H.-W., Lin C.-W., Lee C.-Y., Huang J.-Y., Yang S.-F., Wang K. (2022). The use of androgen deprivation therapy for prostate cancer and its effect on the subsequent dry eye disease: A population-based cohort study. Int. J. Med. Sci..

[26] Agapova O.A., Kaufman P.L., Hernandez M.R. (2006). Androgen receptor and NFkB expression in human normal and glaucomatous optic nerve head astrocytes in vitro and in experimental glaucoma. Exp. Eye Res..

[27] Shiromani S., Bhatnagar K.R., Singh P., Suman S., Meena S., Parveen S. (2022). A study of retinal changes in women with polycystic ovarian syndrome. Indian J. Ophthalmol..

[28] Alpogan O., Donmez E.E., Balık A.Ö., Vural F., Kaplan G. (2021). Effects of testosterone on intraocular pressure, thicknesses of retinal nerve fiber layer, ganglion cell complex, macula and on ocular blood flow in female-to-male transgender persons. Int. Ophthalmol..

[29] Yang S., Zhou J., Li D. (2021). Functions and Diseases of the Retinal Pigment Epithelium. Front. Pharmacol..

[30] Weinreb R.N., Aung T., Medeiros F.A. (2014). The pathophysiology and treatment of glaucoma: A review. JAMA.

[31] Galindez S.M., Keightley A., Koulen P. (2022). Differential distribution of steroid hormone signaling networks in the human choroid-retinal pigment epithelial complex. BMC Ophthalmol..

